# Preeclampsia-Eclampsia Adverse Outcomes Reduction: The Preeclampsia-Eclampsia Checklist

**DOI:** 10.3390/healthcare4020026

**Published:** 2016-05-13

**Authors:** Oroma B. Nwanodi

**Affiliations:** College of Graduate Health Studies, A. T. Still University, Mesa, AZ 85206, USA; o.nwanodi@juno.com; Tel.: +1-314-304-2946

**Keywords:** preeclampsia-eclampsia, prophylaxis, checklist, continuous quality improvement, implementation, patient safety, quality care

## Abstract

Globally, preeclampsia-eclampsia (PE-E) is a major cause of puerperal intensive care unit admission, accounting for up to 10% of maternal deaths. PE-E primary prevention is possible. Antepartum low-dose aspirin prophylaxis, costing USD $10–24 can cut the incidence of PE-E in half. Antepartum low molecular weight heparin combined with low-dose aspirin prophylaxis can cut the incidence of early onset PE-E and fetuses that are small for their gestational age in half. Despite predictive antepartum models for PE-E prophylaxis, said prophylaxis is not routinely provided. Therefore, magnesium sulfate secondary prevention of eclampsia remains the globally recommended intervention. Implementation of a PE-E checklist is a continuous quality improvement (CQI) tool facilitating appropriate antepartum PE-E prophylaxis and maternal care from the first trimester through the postpartum fourth trimester inter-partum interval. A novel clinical PE-E checklist and implementation strategy are presented below. CQI PE-E checklist implementation and appropriate PE-E prophylaxis provides clinicians and healthcare systems an opportunity to achieve Millennium Development Goals 4 and 5, reducing child mortality and improving maternal health. While CQI checklist implementation may be a tedious ongoing process requiring healthcare team resiliency, improved healthcare outcomes are well worth the effort.

## 1. Introduction

Preeclampsia (PE), which globally affects up to 7.6% of pregnancies, including up to 21% of twin pregnancies, is new-onset hypertension from 20 weeks gestation with pulmonary edema, or fetal, renal, hematologic, hepatic, or neurological involvement [1,2,3]. Eclampsia, a seizure occurring in the setting of PE, occurs in 1.4% of pregnancies [1,4,5,6]. Globally, PE-E is the primary cause of puerperal intensive care unit admission and the fourth leading cause of maternal death (5%–10% of maternal deaths). PE-E increases the risk of placental abruption, postpartum hemorrhage, and preterm delivery [1,5]. Severe diastolic hypertension, mean arterial pressures greater than 125 mm Hg, or sustained severe systolic hypertension greater than 170 mm Hg are not requisites for stroke or cerebral hemorrhage in PE-E patients [6]. Globally, intrauterine growth restriction and stillbirth occur in about 30% of cases of PE-E [5]. Early onset PE-E (PE-E occurring before 34 weeks gestation) confers worse morbidity, increasing maternal mortality 20-fold [7]. Therefore, reducing PE-E plays a significant role in achieving Millennium Development Goals 4 and 5, reducing child mortality and improving maternal health [8].

Globally, Africa, Europe, and the western Pacific region (Australia, Japan, the Republic of Korea, New Zealand, and Singapore) have the highest incidence of PE at 4%, 3.8%, and 4.2% [4]. Afghanistan, Pakistan, India and Indonesia all have greater than 1% incidence of eclampsia [4]. In America, African American patients and obese patients with body mass index (BMI) equal to or greater than 30 kg/m^2^ are the primary PE-E stakeholders [6,9]. Each 5 to 7 kg/m^2^ increase in pre-pregnancy BMI doubles PE-E risk [7]. Similarly, in Cameroon, higher BMI is a risk factor for persistent hypertension and proteinuria at three and six months postpartum [10]. Globally, African immigrants in nations other than the United States of America are at higher risk for PE-E [11]. In Canada and the Netherlands, African or Caribbean ancestry is the leading risk for PE-E [11]. In London, England, African ancestry is the one of the leading risk factors for PE-E and stillbirth [11]. Witnessing and treating the sequelae of PE-E’s anticipated adverse effects makes healthcare providers and labor and delivery staff the second victims of PE-E. In acquiring the liability-laden adverse outcome risks of PE-E, and supporting psychologically traumatized staff, healthcare organizations become the third victims of PE-E [6,12].

In high-resource settings, the Fetal Medicine Foundation PE predictive algorithm, comprised of the maternal serum pregnancy-associated placental protein (PAPP-A), placental growth factor (PIGF), maternal uterine artery Doppler pulsatility index (UTPI), mean arterial blood pressure (MAP), and risk factors, is used to predict early and preterm PE [3]. However, PAPP-A, PIGF, and UTPI may be unavailable in low- and medium-resource settings. Once acute preeclampsia-eclampsia (PE-E) is diagnosed, blood pressure risk stratification is insufficient guidance for emergent management [13].

Within the last decade, magnesium sulfate and low-dose aspirin use for PE-E has changed [1]. Minimal risk low-dose aspirin prophylaxis begun at 12–16 weeks gestation gives a 53% relative risk reduction (*RRR*) for PE and a 91% *RRR* for severe PE, at a medication cost of USD $10–24 [1]. Low molecular weight heparin (LMWH) combined with low-dose aspirin can reduce early-onset PE (relative risk (*RR*) 0.54, 95% confidence interval (CI) 0.31–0.92), and the incidence of small for gestational age (SGA) fetuses (*RR* 0.54, 95% CI 0.32–0.91) [14]. The fullPIERS PE-E adverse outcome predictive model, validated in 2011, parallels the Framingham score for cardiovascular risk, as well as the APACHE-IV and SNAP-II scores for adult and neonatal critical care [5]. In the first trimester, Congo Red Dot (CRD) combined with body mass index (BMI), MAP 10 mm Hg higher than mean MAP, a history of PE, and African ancestry increase the accuracy of PE prediction (*p* < 0.001) [3]. The CRD urine test is performed in 30 min at a cost of USD $0.30 [3]. The CRD is undergoing validation and development for first trimester PE prediction and second and third trimester PE diagnosis [3]. Point-of-care options, including mobile health applications, are under development. Other first trimester plasma PE tests under development include the glycosylated fibronectin immunochromatographic strip rapid detection test and the inositol phosphoglycan P-type immunoassay [15].

## 2. Opportunity for Improvement

One potential solution to PE-E morbidity and mortality is to focus exclusively on better inpatient PE-E care by improving adherence to existing treatment protocols. While magnesium sulfate prophylaxis is no longer used for eclampsia prophylaxis at blood pressures less than 160/100, magnesium sulfate remains the most effective eclampsia prophylaxis [16]. As such, rapid PE-E diagnosis and magnesium sulfate secondary prevention of eclampsia remain the globally recommended intervention [17]. A validated antepartum admission PE-E surveillance protocol incorporating the fullPIERS predictive model clinically reduced adverse maternal outcomes significantly (*p* < 0.001) [18].

A second complementary solution is to introduce mandatory PE-E simulations for obstetrics staff, as was done in the United Kingdom, and has been done in the United States at some facilities for obstetric hemorrhage and/or PE-E [19,20,21]. A third complementary solution is a checklist encompassing antepartum screening and PE-E prophylaxis, covering hospitalizations, and continuing through the postpartum fourth trimester inter-partum interval. The checklist can be used for PE-E simulations and can include evidence-based treatment protocols. Checklists are a continuous quality improvement tool.

Continuous quality improvement (CQI) is a means to improve a situation [22]. This entails tracking and analyzing situations, interventions, and outcomes via tools. A given tool may be relevant to one or more processes and phases involved with CQI [23,24]. Historically, bottleneck analysis has been extensively performed for obstetric emergencies [25]. Bottleneck analysis may employ flow charts, cause-and-effect diagrams, Pareto fishbone diagrams, and histograms as part of plan-do-study-act (PDSA) or plan-do-check-act (PDCA) cycles. Once a solution has been implemented, run charts and control charts may be employed to assess change: the study or check stage of PDSA and PDCA cycles [24,26]. Checklists are a newer tool in healthcare CQI than in aviation and other industries. Checklists function as a failsafe, reducing latent failures, breaches, errors, and omissions, including human error [23]. Checklists have been used to prevent events and to control event outcomes [23]. Patient care quality and patient safety are improved by evidence-based checklist adherence [27]. Checklists may offer as much to obstetrics CQI as has bottleneck analysis.

## 3. The Preeclampsia-Eclampsia Checklist

### 3.1. Checklist Development

A Google scholar search was performed for recently published evidence on PE-E and severe maternal hypertension management. Selected evidence is summarized above and below, and is shown in Table 1. Whereas previous algorithms and protocols focus on the inpatient PE-E care, this checklist begins first trimester antepartum care with risk assessment facilitating prophylaxis. Attention was also given to a range of outcome data encompassing American and European end points, increasing research setting relevance for PE-E prophylaxis and treatment outcomes.

### 3.2. The Preeclampsia-Eclampsia Checklist and Strengths

The four-pronged PE-E checklist shown in Table 2 is unique in seeing that the PE-E patient needs care for four trimesters and beyond. The PE-E checklist begins with antepartum prophylaxis, continues through inpatient treatment of a symptomatic PE-E presentation, and culminates in the post-partum course and outcomes summation, leading to fourth trimester inter-pregnancy care. The fourth trimester completes a healthcare cycle, allowing for preconception prophylaxis for subsequent pregnancies. Unlike maternal care bundle algorithms and protocols that are used as reference sheets or pocket flip card sets, the PE-E checklist is designed for annotation, becoming a part of the patient’s medical record. The PE-E checklist places treatment guidelines alongside overall patient treatment steps, which should assist with the improvement of patient outcomes. Preeclampsia-eclampsia–attributed maternal mortality in the United Kingdom declined significantly after the National Institute for Health and Care Excellence 2010 guidance on Hypertension in Pregnancy was promulgated [28]. Outcome summation provides crucial continuity of care data for the immediate six-week postpartum period, the first year postpartum, and any subsequent antepartum period. This is novel, as checklists do not normally play a role in post-event healthcare situations [23].

### 3.3. Checklist Effectiveness

When standard surgical complication care is compared to evidence-based checklists, only 14% of cases are appropriately managed, precipitating additional morbidity (*p* = 0.038) [29]. A retrospective analysis of surgical checklist use in Europe found an association between checklist use and decreased mortality (adjusted odds ratio (*OR*) 0.71, CI 0.58–0.85, *p* < 0.001) [30]. A longitudinal study on the World Health Organization Surgical Checklist implementation in England and Wales found an association between complete three-section (sign-in, time-out, and sign-out) surgical checklist use and reduced postoperative morbidity (*OR* 0.57, CI 0.37–0.87, *p* < 0.01) [31]. However, adherence to the complete three-section surgical checklist use was only 62.1%, reducing the benefits of surgical checklist use [31]. Effectiveness of the PE-E checklist has yet to be determined.

### 3.4. Limitations of the Preeclampsia-Eclampsia Checklist

The PE-E checklist’s comprehensive, four-prong design may be seen as lengthy. The outpatient clinical and inpatient portions require integration. When incompletely adhered to, the PE-E checklist will have reduced effectiveness. The PE-E checklist can only be as effective as health care workers are at implementing the PE-E checklist [31].

## 4. Preeclampsia-Eclampsia Checklist Implementation

Prior to PE-E checklist introduction, a lunch-and-learn or breakfast grand rounds should be held to review the negative effect of PE-E on maternal and fetal outcomes, and on achieving Millennium Development Goals 4 and 5 [5]. The position of national and institutional maternal and fetal outcomes, relative to other high-income nations as well as some middle- and low-income nations should be included in the presentation. For instance, in the United Kingdom, the PE-E maternal mortality rate is 0.38 per 100,000 pregnancies, whereas in California, the rate is 1.6 per 100,000 live births [28]. At a separate lunch-and-learn or breakfast grand rounds, the actual PE-E checklist would be introduced, along with directions on how to use the PE-E checklist. Pre-populated PE-E laboratory tests and ultrasound orders should be created for order simplification.

Separate clinic and hospital PE-E simulations will be run without and then with the checklist [32]. Some healthcare facilities offer or require several obstetric emergency simulations annually, and the PE-E simulation could count as one such simulation [19,20,21]. The expectation is that staff will notice that the simulation is easier to perform with and not without the checklist [32]. This may reinforce the need for checklist use.

Initial uptake among healthcare providers and nursing staff will vary. Checklist integration into existing clinic and hospital procedures, the checklist design, and senior clinician resistance are frequent barriers to surgical checklist implementation [33]. The PE-E checklist implementation may be an opportunity to review and revamp obstetrics clinic and labor and delivery protocols. Each facility should be able to adapt the PE-E checklist. Post-implementation periodic chart review will detect which providers and nurses are or are not using the checklist. Those providers and nurses not using the checklist should be privately contacted, requesting checklist use [18].

Portions of a checklist that are publicly promoted and require the least assigned staff may be the most adhered to [33]. Portions of a checklist that required multiple staff simultaneously are the least adhered to, hence role assignments in the eclampsia sub-list shown in Table 1 [34]. Patients may need to be provided a personal copy of their PE-E checklist to facilitate inpatient use and outcomes data recording.

### Continuous Quality Improvement Assessment

Intervention study for CQI requires data collection and evaluation. Checklist completion forms a five-category independent variable (preventive, antepartum admission and delivery, six weeks postpartum, initial year postpartum, and outcome data) for association with any or all of the outcomes listed in Table 1 as dependent variables [31]. Subpart analysis of the preventive section for adherent patients against outcomes may support low-dose aspirin and LMWH’s PE-E prophylactic efficacy [1,14]. Subpart analysis of the preventive section for non-adherent patients against outcomes data may test the fullPIERS model for PE-E prediction [5]. Parts of the checklist, such as the Institute of Medicine pregnancy weight gain recommendations will change over time as medical knowledge is advanced [35].

## 5. Conclusions

The PE-E checklist provides obstetricians with a minimum-risk means to maintain adherence to evidence-based antepartum prophylaxis and inpatient treatment protocols to improve PE-E outcomes. Postpartum, inter-partum fourth trimester care is also covered in the PE-E checklist. Communication is necessary to ensure that the PE-E checklist is used both in clinic and in hospital, and that outcome data are recorded. Improved communication befits the potential reduction in maternal-fetal morbidity and mortality currently affecting 7.6% of pregnancies globally.

## Figures and Tables

**Table 1:** Selected articles on preeclampsia-eclampsia (PE-E*).*

Reference	Rationale	Methodology	Outcomes	Results
[1]	Low-dose aspirin eligibility.	Mathematical modeling.	Minimum control event rate. Minimum event rate for treatment. Threshold number needed to treat.	Moderately-elevated-risk patients are eligible for low-dose aspirin.
[2]	Management guidelines.	-	-	Synopsis of the 2014 Australia and New Zealand PE-E management guidelines.
[3]	Congo red dot (CRD) urine test is a rapid, affordable diagnostic test.	Prospective cohort.	First, second, and third trimester PE detection.	In the first trimester CRD used alone detects 33.3%, 16.1%, and 20% of early, late, and all PE cases.
[5]	Contextualizes significance of fullPIERS.	-	-	fullPIERS offers PE-E prediction.
[6]	Historic context for maternal severe hypertension care bundle development.	Review article.	Vital signs changes. Systolic blood pressure (SBP), diastolic blood pressure (DBP).	Eclampsia alarm criteria: increases from pregnancy baseline––doubled maternal pulse pressure, SBP by 64 ± 12 mm Hg, or DBP by 31 ± 10 mm Hg.
[7]	Historical context.	-	-	Defined early- and late-onset PE-E.
[9]	California pregnancy-related deaths, 2002–2005. In the US in 1997, maternal mortality rate was 7.7/100,000 live births. By 2009 the rate increased to 17.8/100,000.	Retrospective cohort.	Leading causes of maternal mortality.	Cardiovascular disease, PE-E, hemorrhage, venous thromboembolism, and amniotic fluid embolism accounted for 143 of 207 pregnancy-related maternal deaths from 2002–2005.
[13]	Relevance of continuous quality improvement in women’s healthcare.	-	-	Work with precursors, processes, and indicators to deliver better population health and better healthcare at lower cost.
[14]	Combined antepartum low-dose aspirin and heparin.	Systematic review and meta-analysis.	Incidence of PE, severe PE, early-onset PE, and small for gestational age (SGA) fetuses.	In early-onset PE, low molecular weight heparin in combination with low-dose aspirin offers further reduction of PE and SGA fetuses than use of low-dose aspirin alone.
[16]	Anticonvulsant efficacy for PE-E.	Systematic review of randomized trials of anticonvulsants with or without a placebo control group.	Eclampsia prevention. There was insufficient evidence to compare magnesium sulfate to diazepam, isosorbide, or methyldopa.	Risk of eclampsia is halved by magnesium sulfate, which is more effective than phenytoin and nimodipine. However, magnesium sulfate increases the risk of cesarean delivery when compared to phenytoin.
[18]	Preeclampsia admitting diagnosis patients at a single-tertiary perinatal unit.	24 month pre- and 41 month post-intervention cohort comparison. Intervention was a standardized surveillance protocol.	Any of 17 adverse maternal outcomes and any of seven adverse perinatal or infant outcomes.	Adverse maternal outcomes fell from 5.1% to 0.7%, Fisher *p* < 0.001, odds ratio 0.14, 95% confidence interval 0.04–0.49. Unchanged perinatal outcomes.
[28]	Medical management of severe hypertension	-	-	Severe hypertension treatment protocol.

**Table:** (**A**) First and second trimester record.

Gestation	Signs and Symptoms	Actions	Severe Hypertension Protocol Applies at any Gestation	Postpartum Hemorrhage Protocol
Antepartum visit at 8–16 weeks gestation	□ Body mass index (BMI) > 25 kg/m^2^, if Asian > 23 kg/m^2^ □ Current systolic blood pressure (BP) > than 120 mm Hg: Record BP: --------/------- □ History of miscarriage. □ History of pre-pregnancy hypertension OR chronic kidney disease. □ History of late-onset (34 weeks gestation or later) preeclampsia-eclampsia (PE-E) in a prior pregnancy. □ Diabetes or dyslipidemia.	If yes to any signs and symptoms: □ Order baseline PE-E blood and urine tests. □ Start low-dose aspirin (120–160 mg/day at bedtime. lower doses may be ineffective)––continue until 37 weeks gestation. Check weekly adherence: □ 13, □ 14, □ 15, □ 16, □ 17, □ 18, □ 19, □ 20, □ 21, □ 22, □ 23, □ 24, □ 25, □ 26, □ 27, □ 28, □ 29, □ 30, □ 31, □ 32, □ 33, □ 34, □ 35, □ 36 □ Start calcium supplementation (1.5 g/day)––continue until 37 weeks gestation. Check weekly adherence: □ 13, □ 14, □ 15, □ 16, □ 17, □ 18, □ 19, □ 20, □ 21, □ 22, □ 23, □ 24, □ 25, □ 26, □ 27, □ 28, □ 29, □ 30, □ 31, □ 32, □ 33, □ 34, □ 35, □ 36 □ Ascertain maternal smoking (Ask). □ Yes, smoker □ No, nonsmoker If yes: □ Smoking cessation intervention □ Advise □ Assess □ Assist □ Arrange □ Start First Line Intervention.	Systolic blood pressure (SBP) ≥160 mm Hg Or Diastolic blood pressure (DBP) ≥110 mm Hg for > 15 min. Administer 1) Labetalol 20 mg iv. over 2 min, except if asthmatic or in heart failure. a) If BP still severe in 10 min, give 40 mg labetalol iv. b) If BP still severe in 10 min, give 60 mg labetalol iv. c) If BP still severe in 10 min, give 80 mg labetalol iv. d) If BP still severe in 10 min, consultation per protocol.	*Insert institution Postpartum Hemorrhage protocol here*. Check for: Transfusion availability □ Platelets □ Fresh frozen plasma □ Cryoprecipitate □ Packed red blood cells Check for: □ Cell saver applicability
Antepartum visit at 12–16 weeks gestation	□ History of antiphospholipid antibody syndrome. □ History of early-onset PE-E (onset at before 34 weeks gestation) in a prior pregnancy. □ History of small for gestational age fetus inconsistent with parentage.	□ Start unfractionated or low-molecular weight heparin. Check weekly adherence: □ 13, □ 14, □ 15, □ 16, □ 17, □ 18, □ 19, □ 20, □ 21, □ 22, □ 23, □ 24, □ 25, □ 26, □ 27, □ 28, □ 29, □ 30, □ 31, □ 32, □ 33, □ 34, □ 35, □ 36 □ Use low-dose aspirin and calcium. Check □ above. □ Ascertain maternal smoking (Ask). □ Yes, smoker □ No, nonsmoker If yes: □ Smoking cessation intervention □ Advise □ Assess □ Assist □ Arrange	Start hydralazine. Do not exceed 300 mg labetalol/24 h. 2) Hydralazine 5 mg iv. First line if pulse <60 bpm. Can give 250 mL normal saline bolus to reduce reflex tachycardia. a) If BP still severe in 20 min, give 10 mg hydralazine iv. b) If BP still severe in 20 min, give 20 mg labetalol iv. c) If BP still severe in 20 min, Give 40 mg labetalol iv.	Institute of Medicine weight gain guide Pre-pregnancy Total BMI Weight gain lbs <18.5 28–40 18.5–24.9 25–35 25.0–29.9 15–25 ≥30.0 11–20
Antepartum visit after 16 weeks	□ Singleton gestation. □ Multiple gestation.	If multiple gestation: □ Perinatology/maternal-fetal-medicine consultation ordered. □ Perinatology/maternal-fetal-medicine consultation performed.	Consultation per protocol. 3) Oral nifedipine up to 90 mg daily.	Second and Third Trimester Weight Gain Rate BMI lbs/week <18.5–24.9 1.0 25–29.9 0.6
Before 24 weeks	□ Severe PE-E.	□ Consider pregnancy termination due to greater than 80% perinatal mortality and up to 71% maternal morbidity (Lowe et al., 2015). □ Peridelivery thromboprophylaxis.	Short-acting is preferred. 4) Oral labetalol 200 mg.	1^st^ Trimester Weight Gain All BMIs 1.1–4.4 lbs
□ Excessive	Interval weight gain	□ Revisit First Line Intervention		

**Table:** (**B**) Second and third trimester record.

Gestation	Signs and Symptoms	Actions	Magnesium Sulfate Protocol	Initial Seizure Protocol
24–36 weeks	Symptomatic preeclampsia: □ Headache □ Visual changes □ Nausea □ Right upper quadrant, or Epigastric pain, or Chest pain.	□ Order PE-E blood and urine tests. □ Order fetal ultrasound including □ complete biophysical profile, □ umbilical artery dopplers. □ Administer antenatal corticosteroids for fetal lung maturity. □ Administer magnesium sulfate for fetal neuroprotection, maternal blood pressure lowering, and seizure prophylaxis. □ Order peri-delivery thromboprophylaxis. □ Order consultations: □ Neonatology □ Maternal-Fetal-Medicine □ Request neonatology to be at delivery.	Initial bolus 4–6 g in 100 mL normal saline iv. over 20 min. □ 4 g bolus OR □ 6 g bolus Maintenance 1–2 g iv./h □ 1 g/h––no serum monitoring OR □ 2 g/h––order serum monitoring OR Alternate im loading dose is 10 g in 50% solution total, as 5 g per buttock in 1–2 ml plain lidocaine: □ Left buttock □ Right buttock	□ Initial 4–6 g magnesium sulfate bolus over 15–20 min. Maintain BP <160/100 mm Hg Second line therapies: □ Diazepam 5–10 mg iv. Every 5–10 min, maximum 30 mg total. Or □ Phenytoin: pharmacy dosing □ MRI for neuroimaging [do not delay delivery for]. OR □ CT
	Antepartum Admission	□ Order Admission day + 1 PE-E blood tests. □ Order daily external fetal monitoring. □ Order Monday and Thursday PE-E blood tests, □ ultrasound for amniotic fluid index, □ ultrasound for umbilical artery Doppler. □ Order once weekly spot urine protein / creatinine ratio or 24 h urine total protein and creatinine clearance. □ Order complete fetal ultrasound every 14 days	For hypermagnesemia Administer calcium gluconate 1 g as 10 ml of 10% solution iv over 1–2 min.	
Deliver if any of:	□ Eclamptic seizure, OsR □ hemolysis, elevated liver enzymes, and low platelets (HELLP), OR □ Disseminated intravascular coagulation (DIC), OR □ Intrauterine demise, OR □ Placental abruption, OR □ Uncontrollable hypertension, OR □ Pulmonary edema, OR □ Non-reassuring fetal status.	Immediate delivery. HELLP has a 6.3% maternal mortality, increased risk of placental abruption, and postpartum hemorrhage. □ Administer magnesium sulfate for fetal neuroprotection, maternal blood pressure lowering, and seizure prophylaxis. □ Order peridelivery thromboprophylaxis □ Order PE-E blood and urine tests, □ fetal ultrasound including □ complete biophysical profile, □ umbilical artery dopplers. □ Order platelet transfusion if < 50 × 10^9 ^/L □ Order consultations: □ Neonatology □ Maternal-Fetal-Medicine	Eclampsia Rapid Response □ Appoint a leader. □ Appoint a recorder. □ Appoint primary nurse. □ Appoint secondary personnel. □ Protect airway. □ Secure patient in bed, rails up. □ Place patient in left lateral decubitus position. □ Secure i.v. access and labs. □ 100% non-rebreather O2. □ Bag-mask ventilation. □ Suction airway if necessary.	Recurrent Seizure Protocol □ Additional 2–4 g magnesium sulfate bolus over 10 min.

**Table:** (**C**) Term and fourth trimester record.

Gestation	Action	
After 36 weeks	□ Immediate delivery. □ Administer magnesium sulfate for maternal blood pressure lowering, and seizure prophylaxis. □ Order PE-E blood and urine tests. □ Order pre-delivery fetal ultrasound including, □ complete biophysical profile □ umbilical artery dopplers. □ Request neonatology at delivery.	
Post-delivery Day (PPD) 1	□ Order PE-E blood tests. □ Continue magnesium sulfate for 24 h post-delivery.	
Post-delivery Day (PPD) 2	□ Order PE-E blood tests. Ambulate patient four times daily: □ PPD2 □ PPD3 □ PPD4 □PPD5 □ PPD6 Patient sits up in chair for meals: □ PPD2 □ PPD3 □ PPD4 □PPD5 □ PPD6	
Post-delivery Day (PPD) 7	□ Order PE-E blood tests. Ambulate patient four times daily: □ PPD7 □ PPD8 □ PPD9 □PPD10 □ PPD11 Patient sits up in chair for meals: □ PPD7 □ PPD8 □ PPD9 □PPD10 □ PPD11	
Post-delivery Week 6	□ Measure and record clinical blood pressure: ______/_____ □ Measure and record clinical weight: _______ □ Measure and record clinical height: ________ □ Record body mass index (BMI) : _______ □ First Line Intervention for BMI > 25 kg/m^2^. If Asian, BMI > 23 kg/m^2^.	First Line Intervention □ Specialist diet and activity history assessment □ Counseling referral to Registered Dietitian or Nutritionist. □ Exercise prescription and referral to Physical Trainer.
Post-delivery Weeks 16–46	Clinical blood pressure monitoring every 10 weeks. Record values: □ Week 16 □ Week 26 □ Week 36 □ Week 46 _____/____ ____/____ ____/____ ____/____ Clinical weight monitoring every 10 weeks. Record values: □ Week 16 □ Week 26 □ Week 36 □ Week 46 _________ _________ _________ ________ Clinical body mass index monitoring every 10 weeks. Record values: □ Week 16 □ Week 26 □ Week 36 □ Week 46 _________ _________ _________ _________ □ First Line Intervention for BMI > 25 kg/m^2^. If Asian, BMI > 23 kg/m^2^. □ Annual lipid panel and blood glucose. First Line Intervention if abnormal.	

**Table:** (**D**) Outcome measures.

Maternal Outcome Measures	Perinatal Outcome Measures
□ Maternal death □ Hepatic failure □ hepatic hematoma □ hepatic rupture □ Glasgow coma score < 13 □ Stroke	□ Stillbirth □ Neonatal death □ Infant death
□ Two or more seizures □ Cortical blindness □ Positive inotrope support □ Myocardial infarction □ Third intravenous antihypertensive used □ Renal dialysis □ Renal transplantation □ 50% FIO2 for >1 h □ Pulmonary edema □ Pneumonia □ Intubation □ Postpartum hemorrhage. If checked choose: □ Medically managed □ Balloon tamponade □ Dilation and curettage □ Hysterectomy □ Uterine artery embolization □ Transfusion of 10 or more units of blood Products □ Intensive care unit admission Record highest BP mm Hg in first 24 h of admission: Systolic BP: _______ Diastolic BP:_______ Pulse pressure:_______ Mean arterial pressure:_______ Record history urine protein in first 24 h of admission: _______grams Record highest Aspartate transaminase in first 24 h of admission: ________ Record lowest platelet count in first 24 h of admission: ________ × 10^9^/L □ Cesarean delivery. If checked choose all that apply for anesthesia: □ Spinal □ Epidural □ General □ Vaginal delivery □ Labor induction □ Length of labor □ Length of hospitalization	□ Bronchopulmonary dysplasia □ Necrotizing enterocolitis □ Grade III/IV intraventricular hemorrhage □ Cystic periventricular leukomalacia □ Stage 3–5 retinopathy of prematurity □ In special care nursery for more than 10 days: Record the number of days: ______days Record gestational age at delivery: _____weeks Record birth weight: _______grams □ Small for gestational age Record birth weight percentile _____ Record 0, 1, 5, and 10 min APGAR score _______, _______, ________, _______

im = intramuscular; iv = intravenous; BP = blood pressure; PE-E blood tests = Complete blood count (CBC), international normalized ratio (INR), activated partial thromboplastin time (APTT), fibrinogen, comprehensive metabolic panel (CMP), and uric acid; PE-E urine tests = Congo red dot, and/or spot urine protein/creatinine ratio, or 24 h total urine protein and creatinine clearance; PPD = Post-delivery day.

## Abbreviations

The following abbreviations are used in this manuscript:

APACHE-IV	Acute physiology and chronic health evaluation version IV
BMI	Body mass index
CQI	Continuous quality improvement
fullPIERS	Predictive model for maternal outcomes within 48 h of preeclampsia admission based on the pre-eclampsia integrated estimate of risk
LMWH	Low molecular weight heparin
MAP	Mean arterial blood pressure
OR	Odds ratio
PAPP-A	Maternal serum pregnancy-associated placental protein
PDCA	Plan-do-check-act
PDSA	Plan-do-study-act
PE	Preeclampsia
PE-E	Preeclampsia-eclampsia
PIGF	Placental growth factor
RRR	Relative risk reduction
SGA	Small for gestational age
SNAP-II	Simplified newborn illness severity and mortality risk scores
UTPI	Maternal uterine artery doppler pulsatility index
